# Which fascial plane block is right for my patient? A practical decision-making algorithm for abdominal surgery

**DOI:** 10.1186/s44158-026-00400-z

**Published:** 2026-05-11

**Authors:** Ellile Sultan, Matthew A. Kosasih, Amit Pawa, Barbara Versyck, Edward R. Mariano

**Affiliations:** 1 Stanford University School of Medicine, Stanford, CA USA; 2 Guy’s and St. Thomas’ NHS Foundation Trust, London, UK; 3 Cleveland Clinic Lerner College of Medicine, Case Western Reserve University School of Medicine, Cleveland, OH USA; 4 AZ Turnhout, Turnhout, Antwerp Belgium; 5 Veterans Affairs Palo Alto Health Care System, Palo Alto, CA USA

**Keywords:** Abdominal surgery, Acute pain management, Clinical decision-making, Fascial plane block, Regional analgesia, Truncal block

## Abstract

**Background:**

Optimizing postoperative pain management is central to successful recovery following abdominal surgery and is a key component of enhanced recovery protocols. While multimodal analgesia is considered standard practice, selecting the most appropriate regional analgesic technique remains challenging, especially when neuraxial analgesia is contraindicated or undesirable. Ultrasound-guided abdominal fascial plane blocks have emerged as valuable alternatives, offering targeted somatic analgesia with favorable safety profiles. However, the growing number of available techniques has created uncertainty regarding optimal block selection for specific abdominal surgeries.

**Clinical decision tool:**

Our clinical decision tool aims to complement existing guidelines regarding fascial plane blocks with a simple algorithm to help the general anesthesiologist choose an appropriate fascial plane block for specific abdominal surgeries. We have also considered real-world factors such as ultrasound availability and institutional support for continuous catheters. Within this review, we discuss the current evidence and technical performance for the following procedures: transversus abdominis plane block, rectus sheath block, external oblique intercostal plane block, ilioinguinal and iliohypogastric nerve block, and quadratus lumborum block. Consistent with the Plan A blocks framework, this decision-making algorithm applies updated nomenclature and consideration of practical factors that influence choice of block by the general anesthesiologist when performing regional analgesia at the point of care.

**Conclusion:**

Abdominal fascial plane blocks are versatile and low-risk additions to a multimodal analgesic regimen for abdominal surgery. Our structured, site-specific, decision-making framework can assist physicians in selecting the most appropriate fascial plane block for abdominal surgery while accounting for patient, surgical, and institutional factors. Such an approach supports individualized analgesic planning and may enhance postoperative recovery, particularly when neuraxial techniques are unsuitable or unavailable.

## Background

The postoperative experience and successful recovery from surgery have garnered increasing attention and resources in recent years. Through initiatives to develop procedure-specific protocols focused on enhancing recovery, particularly for abdominal and pelvic surgery, defining and delivering successful surgical recovery are prioritized in many institutions worldwide [1].

Individualized postoperative pain prediction would be ideal in selecting the most appropriate analgesic plan, and this may become reality some day. Exciting predictive models are emerging, such as the Peri-operative Quality Improvement Programme [2] and tools powered by artificial intelligence; however, we are not at the stage of executing a truly personalized analgesic approach yet [3].

Modern perioperative pain management principles include routine use of multimodal analgesia regardless of the type of surgery [4]. Multimodal analgesia starts with ‘basic’ components such as nonpharmacologic therapies and systemic nonopioid medications which can be used for all patients, barring any contraindications [5]. Local or regional analgesia is an important part of the multimodal analgesic regimen [6], and all patients should receive at a minimum infiltration of local anesthetic by the surgeon. For abdominal and pelvic surgeries, regional analgesic techniques have well-established efficacy, and neuraxial techniques can provide visceral pain coverage [7].

Success of enhanced recovery protocols is often measured by a shorter duration of hospital stay and the avoidance of postoperative complications. The traditional gold standard in pain management for abdominal surgery, thoracic epidural analgesia (TEA), can sometimes be at odds with these goals due to non-insignificant issues with hypotension, mobilization, and urinary retention [8]. However, the superior somatic and visceral coverage from TEA has meant that, for surgeries with higher expected postoperative pain such as cytoreductive surgery with hyperthermic intraperitoneal chemotherapy, TEA remains strongly indicated in enhanced recovery protocols [9].

For patients undergoing abdominal or pelvic surgery in which a neuraxial technique is contraindicated (e.g., anticoagulation) [10] or not preferred, a variety of peripheral options have emerged in the form of ultrasound-guided fascial plane blocks [11]. Generally, abdominal fascial plane blocks are considered lower-risk procedures that can be safely performed in patients with pharmacologic anticoagulation or coagulopathy given the majority of these blocks are superficial and distant from major blood vessels [12]. However, most abdominal fascial plane blocks do not provide visceral coverage, as visceral afferent nociceptive fibers travel with the sympathetic nervous system back to the spinal cord and are far away from the sites of local anesthetic injection [7, 13]. While neuraxial and paraspinal techniques have greater potential to cover visceral pain, they carry higher risk of critical bleeding [12], may be more technically challenging, and cannot be performed in the anesthetized patient positioned supine for abdominal or pelvic surgery. Fascial plane blocks have effectively occupied the middle ground between neuraxial techniques and no block at all, and physicians can select the best option based on indication, logistical factors, and the balance of benefits versus potential risks to incorporate within a multimodal analgesic plan for their patients.

Originally introduced as blind, landmark-based techniques [14], fascial plane blocks are now routinely performed with ultrasound guidance. The complete mechanism of action of fascial plane blocks is still not completely understood [13] but is likely multifactorial. End targets include groups of nerves and plexi that travel within the fascial planes [15] as well as emerging evidence of nerve endings within fascial planes [16], both of which can be blocked by local anesthetics. There may also be direct muscle relaxation within the fascial plane resulting in analgesia [17] as well as detectable serum levels of local anesthetics after abdominal fascial plane blocks which may contribute to systemic analgesic effects or other modes of action [18]. The American Society of Anesthesiologists (ASA) recently published practice guidelines on the use of regional analgesia for truncal surgeries and gave strong recommendations favoring fascial plane blocks for both open and minimally invasive abdominal surgeries based on a systematic review and meta-analysis [6]. While the recommendations supporting fascial plane blocks were based on at least moderate strength of evidence, they do not specify which block technique to perform for a particular indication.

The remainder of this review will focus on the introduction of a novel decision-making guide for the general anesthesiologist when choosing among the many ultrasound-guided fascial-plane block options for their patients who are undergoing abdominal surgery.

## Clinical decision tool

**Fig. 1:**
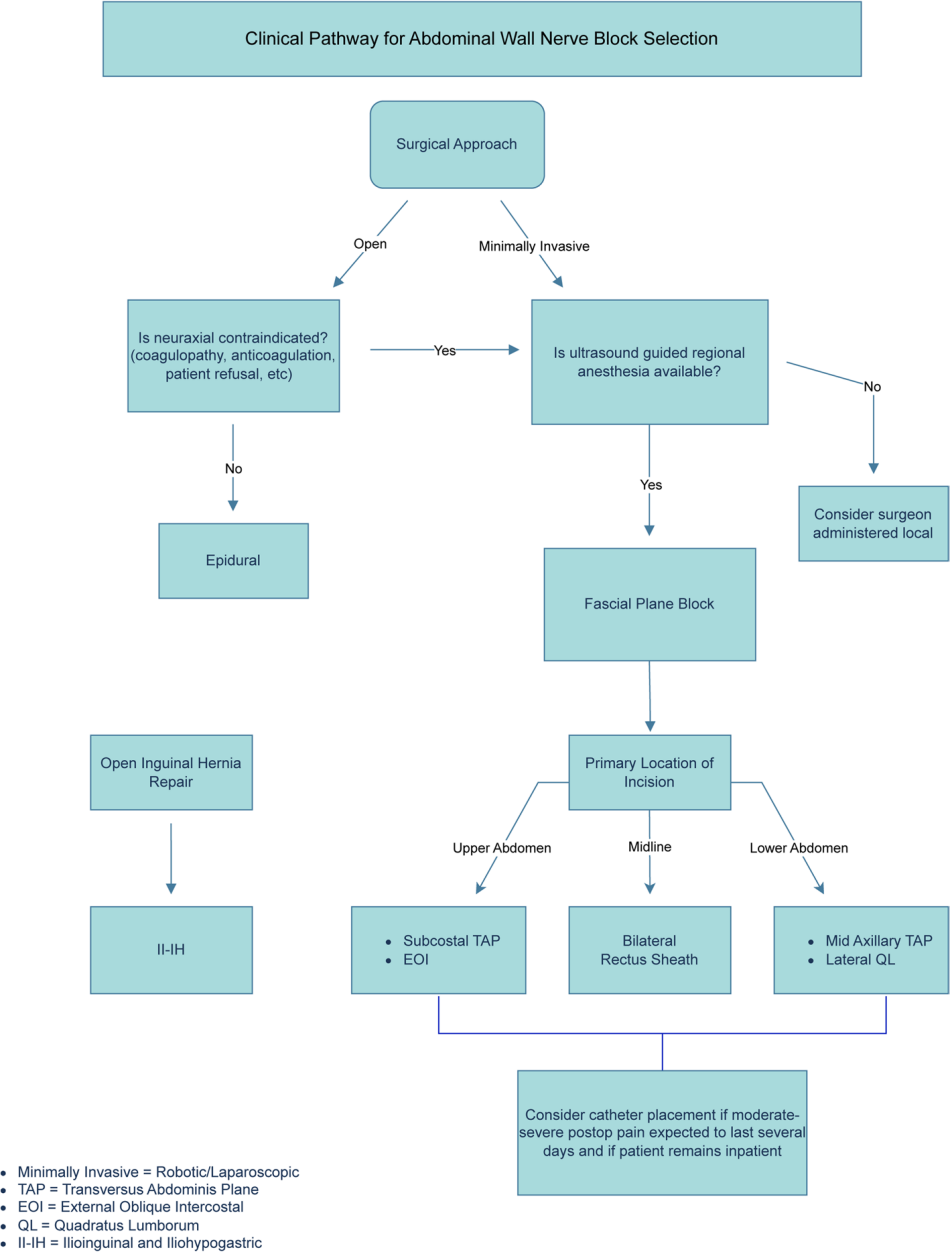
Clinical decision-making tool for selecting the most appropriate fascial plane block for patients undergoing abdominal surgery

The authors developed a novel decision-making pathway (Fig. 1) to help physicians decide which fascial plane block (if any) to perform based on clinical indication. In line with the Plan A blocks framework [19, 20], this tool focuses on a ‘few blocks for the many’ with the goal of increasing the accessibility of regional analgesia for all patients. The creation and implementation of this tool occurred at a single center and was based on currently available evidence and local expert consensus with an emphasis on simplicity and practical applicability for the general anesthesiologist (not the regional anesthesia fellowship-trained physician expert working at a high-complexity academic center). A key assumption made in the development of this tool was that fascial plane blocks for abdominal surgery would be performed in the operating room with the patient under general anesthesia, either after anesthetic induction or before emergence. Accordingly, paraspinal blocks (e.g., paravertebral, erector spinae plane) were not considered due to the need to reposition the patient and the potential negative effects on efficiency and patient safety. Prior to implementation, this decision-making tool was reviewed and iteratively revised by consultant anesthesiologists specializing in regional anesthesia and acute pain medicine as well as consultant surgeons involved in the procedures covered. An audit of eligible cases over a 3-month period in the year following implementation demonstrates 96% institutional adherence to the algorithm (unpublished data presented at the 2026 American Society of Regional Anesthesia and Pain Medicine spring annual meeting).

Although the algorithm is focused on regional analgesic techniques, we wish to emphasize that all patients should be provided multimodal analgesia in accordance with recent multi-society guidance [4] and that our pathway is intended to serve the majority of patients undergoing abdominal surgery who can be expected to experience at least moderate postoperative pain. Patients who are at risk for higher levels of pain (e.g., known chronic pain or substance use disorders) may need more nuanced and personalized analgesic plans.

All branches of the pathway lead to a fascial plane block recommendation in accordance with recent ASA guidelines [6]. Our pathway also incorporates surgeon-administered local anesthesia when regional analgesia is not available. We have included neuraxial as an option for major open abdominal surgery, despite its decreasing use [21] and growing support for fascial plane blocks, because of the superior visceral pain coverage and continued inclusion in major open hepatobiliary guidelines [22, 23].

One important ‘open surgery’ exception is inguinal hernia repair. While it is an open procedure, it involves a relatively smaller incision, is primarily limited to the abdominal wall, and is generally associated with less myofascial trauma than intraperitoneal procedures. Ilioinguinal and iliohypogastric nerve blocks may provide adequate analgesia when performed by the anesthesiologist or surgeon.

**Fig. 2:**
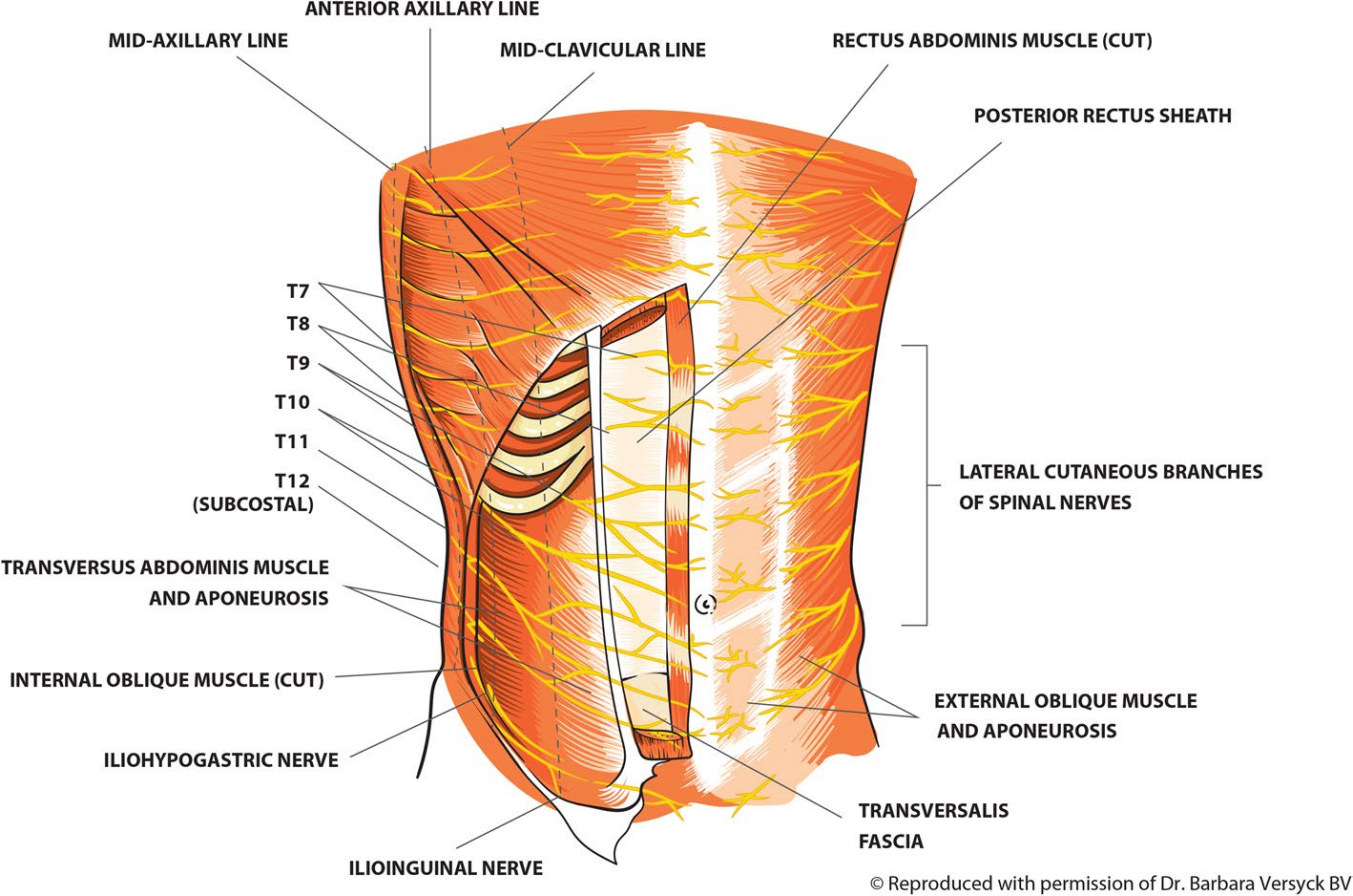
Abdominal wall anatomy and innervation

**Fig. 3:**
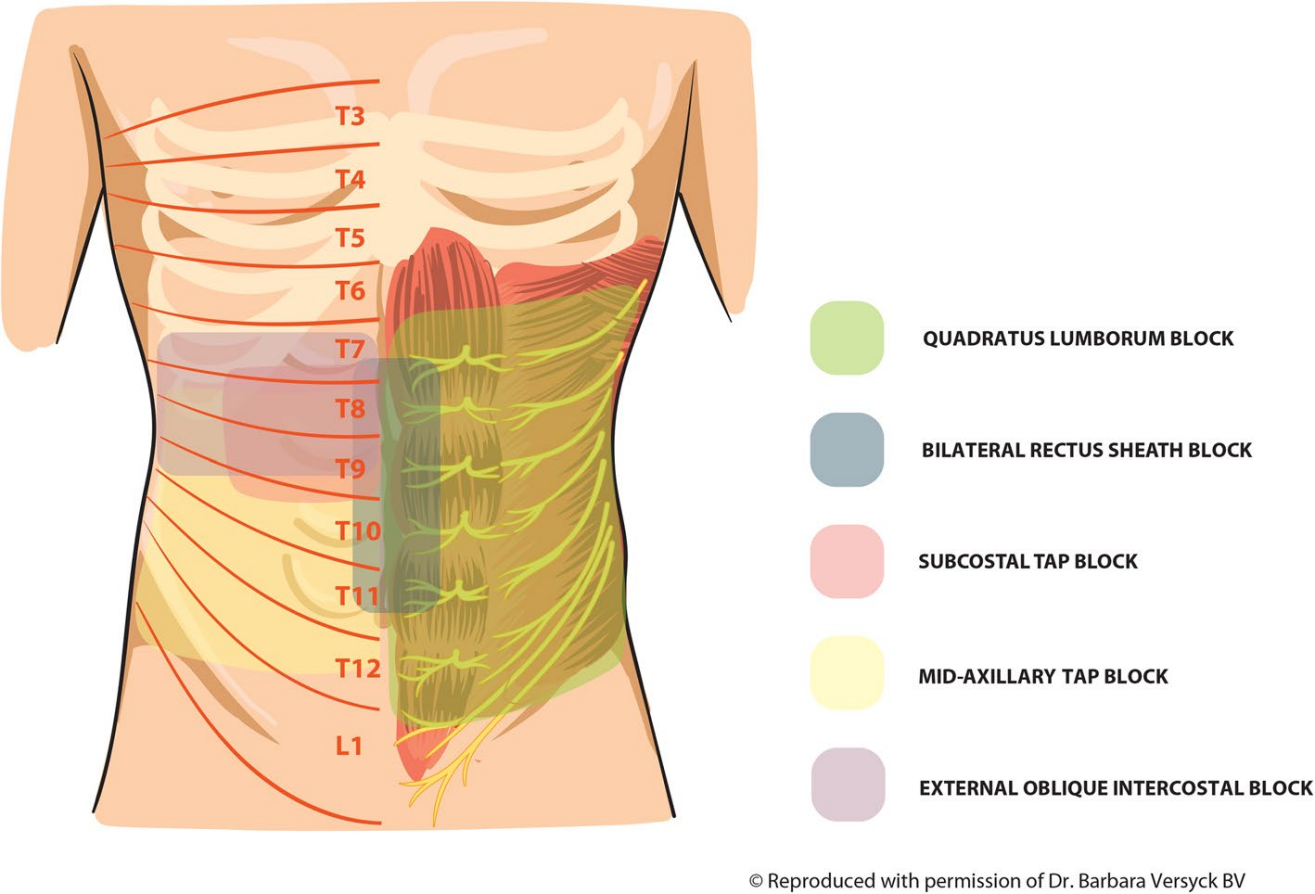
Select fascial plane blocks that have been shown to cover the abdominal wall. Shown in the illustration is the largest potential area of analgesic coverage for each block, and interindividual variation (due to patient, performer, or other factors) should be expected. TAP, transversus abdominis plane

However, the main purpose of this pathway is to help the general anesthesiologist decide between the various fascial plane blocks by utilizing the site of the primary surgical incision as the indicator. The muscular anatomy of the abdominal wall and innervation are shown in Fig. 2, and a graphical representation of potential areas covered by specific fascial plane block techniques is shown in Fig. 3.

Surgical factors which are associated with greater levels of pain include midline incisions; laparoscopic approaches with four ports; scalpel rather than diathermy cutting; higher pressure pneumoperitoneum (> 12 mmHg); passive (rather than active) desufflation of the peritoneum; and wound size more than 10 cm [24, 25]. Some patient factors that are predictive of higher levels of postoperative pain are female sex, younger age, high preoperative pain, and pain catastrophizing [25]. If these factors are present, the anesthesiologist should consider additional elements of multimodal analgesia, utilizing longer-acting local anesthetics when performing a regional technique, or placing indwelling catheters for postoperative local anesthetic infusion or intermittent bolus if available.

A few additional nerve block factors must also be considered. These include timing of block (preinduction, preemergence, or postoperatively); the type of pain coverage needed (somatic versus visceral); technical complexity of the procedure; comfort/training level of the proceduralist; institutional support (e.g., catheters, pumps, and other follow-up care); and patient-specific indications or contraindications (e.g., coagulopathy, nearby infection, patient refusal).

The optimal timing for abdominal fascial plane blocks in the perioperative setting is guided by institutional workflow; however, technical challenges associated with block placement must be considered. Specifically, performing a block preemergence or postoperatively in the postanesthesia care unit may be more technically challenging due to surgical disruption of the myofascial planes and entrapment of air, which can compromise ultrasound visualization. This challenge is especially relevant following open surgical procedures or very long minimally invasive surgeries. Finally, when continuous catheter analgesia is planned, block selection and placement during the preoperative or post-induction period require ensuring the catheters are situated far enough away from the surgical site to mitigate the risk of accidental removal or dislodgement.

Each of the included fascial plane blocks is discussed in more detail below.

## Fascial plane blocks

### Transversus abdominis plane block

The transversus abdominis plane (TAP) block targets the distal branches of the ventral rami of the T6–L1 thoracolumbar nerves that travel between the internal oblique and the transversus abdominis muscles (Fig. 2) [26]. These nerves supply the anterior abdominal muscles and the overlying layers. At the linea semilunaris, each of these thoracolumbar nerves gives off an anterior cutaneous branch which supplies the rectus abdominis muscle and the skin around the midline [27].

The original description of this block was a landmark technique utilizing the insertion of the latissimus dorsi muscle onto the iliac crest to identify the triangle of Petit. Insertion of a block needle anterior to this point and just superior to the iliac crest allowed the proceduralist to encounter a fascial ‘pop’ as the internal oblique fascia was breached [14].

There are multiple fascial layers and sublayers within the abdominal wall, and they form strong connections at various anatomical sites [28]. These connections can impede the free movement of local anesthetics throughout the plane; therefore, alternative TAP approaches have been developed following the original description of this block in 2001. These have been recently rationalized down to two TAP block approaches through an international consensus project: subcostal TAP (upper abdominal wall coverage) and midaxillary TAP (lower abdominal wall coverage) (Fig. 3) [11].

#### Subcostal TAP

Subcostal TAP blocks may be used for any upper abdominal surgery (e.g., cholecystectomy, partial liver resection, splenectomy or supraumbilical hernia repair) (Fig. 3).

#### Block performance

With the patient supine, a high-frequency, linear probe is positioned inferiorly and in line with the costal margin at the xiphisternum. The probe is translated laterally, along the inferior border of the costal margin to view the origin of the transversus abdominis muscle posterior to the rectus abdominis muscle. A needle is inserted from medial to lateral to access the fascial plane between the transversus abdominis muscle and the internal oblique muscle or aponeurosis. Coverage of the most cephalad margin may be variable depending on how close to the linea semilunaris the local anesthetic is administered [29].

### Midaxillary TAP

Midaxillary TAP blocks may be used for any abdominal surgery with lower-quadrant incisions (e.g., hysterectomy, Caesarean delivery, colectomy) (Fig. 3).

#### Block performance

With the patient supine, a high-frequency linear probe is placed in a transverse orientation at the midaxillary line at the level of the umbilicus or midway between the subcostal margin and iliac crest. The needle is directed in-plane from anterior to posterior, and local anesthetic is deposited in the fascial plane between the internal oblique and the transversus abdominis muscles.

#### Clinical pearls and evidence

TAP blocks have the potential to be used for any abdominal surgery. Although the overall utility of the TAP block has been questioned [30], analyses of specific surgical cohorts have shown measurable reductions in pain intensity and rescue opioid consumption despite many sources of heterogeneity [31, 32]. It is important for anesthesiologists to pay close attention to the location of the surgical incision(s) before deciding if a TAP block is indicated and which one to do.

Surgeons may also directly infiltrate local anesthetic into the TAP; when performed correctly, this can produce analgesic benefit and reduced morphine consumption in the short term [33].

Visceral pain is not covered with a TAP block, so surgeries with a high visceral component (e.g., extensive pelvic tumor surgery) may have limited benefit [34]. In the Cesarean delivery population, the benefits of TAP block are masked by intrathecal opioids; however, this may be duration-dependent as the use of liposomal bupivacaine for TAP block has been shown to improve recovery quality [35].

Beyond these considerations, there is evidence that TAP blocks combined with multimodal analgesia are superior to multimodal analgesia alone and may also contribute to improved surgical outcomes in certain groups (e.g., return of bowel function after laparoscopic cholecystectomy) [36].

#### Rectus sheath block

The development of the rectus sheath block precedes the TAP block and was initially thought to anesthetize the whole of the anterior abdominal wall [37]. However, clinically the rectus sheath block typically covers the anterior cutaneous branches of the thoracolumbar nerves T6–L1 which cover the rectus abdominis muscle and the overlying skin. When performed bilaterally, this block provides coverage for surgeries with a midline incision (e.g., exploratory laparotomy, ventral/umbilical hernia) (Fig. 3). The linea alba divides the two rectus abdominis muscles and is tightly adherent to the rectus sheath; therefore, bilateral blocks should be performed to cover both sides of the midline.

#### Block performance

With the patient supine, a high-frequency linear probe is placed in the midline, just superior to the umbilicus in transverse orientation. The central linea alba is identified and the ‘butterfly-wing' appearance of the dual rectus abdominis muscles is noted. The probe is translated laterally to identify the lateral edge of the rectus muscle. The needle is inserted from lateral to medial into the space between the posterior rectus sheath and the rectus abdominis muscle, if below the arcuate line. Above the arcuate line, the transversus abdominis muscle extends medially posterior to the rectus abdominis muscle; injection of local anesthetic between these two muscles will also extend into the rectus sheath.

#### Clinical pearls and evidence

Of all the abdominal surgical incisions, midline incisions are associated with the greatest degree of postoperative pain and pulmonary complications [38]; therefore, supplemental analgesia with bilateral rectus sheath blocks offers clinical benefits. In settings that can offer patients continuous rectus sheath blocks, patients can have significant reductions in pain scores and 24-h opioid consumption [39]. There is some evidence that multiple rectus sheath injections (bilateral above and below the umbilicus) may be required to provide full coverage from the xiphisternum to the pubis [40].

Surgeons may also perform rectus sheath blocks directly at the time of closure. These are relatively easy to perform and have reported efficacy [41]. This may be a good option for patients in whom a planned laparoscopic procedure is converted to open.

#### External oblique intercostal plane block

The external oblique intercostal (EOI) plane block was recently developed to target the upper abdominal wall similar to a subcostal TAP block (Fig. 3) [42]. This region is innervated by the distal branches of T6–T8 (Fig. 2) [27].

#### Block performance

With the patient supine and ipsilateral arm abducted, a high-frequency linear probe in the sagittal orientation is placed between the midclavicular and the anterior axillary line to identify the sixth rib. The reference points of the seventh rib found in line with the xiphisternum and/or the 10th rib found at the costal margin can be used. The cranial end of the probe is rotated medially to obtain a sagittal-oblique view and local anesthetic is delivered between the ribs and the external oblique muscle or aponeurosis (Fig. 2). The local anesthetic between the external oblique and intercostal muscles can be visualized spreading to the adjoining rib interspaces.

#### Clinical pearls and evidence

Despite being a newer approach, there is growing evidence supporting the use of EOI plane blocks for laparoscopic sleeve gastrectomy, laparoscopic and open hepatectomy, and laparoscopic cholecystectomy [43–45]. For these surgeries, EOI blocks contribute to decreased 24-h opioid consumption; for patients undergoing hepatectomy, this benefit may extend to 48 h postoperatively [45]. Compared to a subcostal TAP block, the EOI plane block may be less affected by upper abdominal wall trauma from dissection and/or incisions when performed after surgical closure.

As the EOI block is performed at a site further distant from the surgical incision(s), this technique may be performed preoperatively and with catheter placement to provide prolonged, continuous analgesia after surgery.

#### Ilioinguinal and iliohypogastric (ILIH) nerve blocks

Both ilioinguinal and iliohypogastric nerves arise from the lumbar plexus, most commonly the L1 branch, travel in the TAP similar to other thoracolumbar nerves (Fig. 2), and are found in close proximity to each other and the deep circumflex iliac artery. Together, these nerves innervate the skin of the groin, anteromedial thigh, suprapubic region, and inferior portion of the three abdominal wall muscles (external and internal oblique and transversus abdominis).

#### Block performance

With the patient supine, a high-frequency linear probe is placed over the anterior superior iliac spine (ASIS) in an oblique orientation with the lateral side of the probe over the ASIS and the medial side of the probe aiming towards the umbilicus. The plane between the internal oblique and the transversus abdominis muscle is identified and local anesthetic is infiltrated between them to surround the neurovascular bundle [46]. The deep circumflex iliac artery may be visualized with color Doppler in the same plane as the nerves.

#### Clinical pearls and evidence

ILIH nerve blocks can be used for postoperative analgesia for inguinal hernia repair and Caesarean delivery or to treat postherniorrhaphy chronic pain syndromes. When combined with local anesthetic infiltration or TAP block, ILIH block can be used as surgical anesthesia for open inguinal hernia repair and, when compared to spinal anesthesia, can lead to faster discharge home after outpatient surgery [47]. As with rectus sheath blocks, intraoperative ILIH blocks may be performed directly by surgeons into the open inguinal canal with good outcomes which are comparable to landmark- and ultrasound-guided techniques [46]. Comparison of landmark-based to ultrasound-guided ILIH techniques shows fewer block-related complications with ultrasound, similar effectiveness in adults, and improved effectiveness in pediatric patients [48]. For Caesarean delivery, single-injection bilateral ILIH nerve blocks appear to be noninferior to single-injection bilateral TAP blocks [49]. The potential benefits of catheter-based techniques have not yet been fully explored.

#### Quadratus lumborum blocks

The quadratus lumborum (QL) block was described by Blanco in 2007 [50] and makes use of the unique position of the QL muscle, which is posterior to the psoas major muscle and adjacent to the origin of the TAP.

Over time, the QL block has developed into 3 variants: lateral, posterior and anterior [11]. The name refers to the location of the local anesthetic deposition relative to the QL muscle.

#### Block performance

The needle insertion site for QL block tends to be more posterior than the midaxillary TAP block. Suggested patient positions include: lateral decubitus, with the side to be blocked nondependent; sitting; or prone. Prone positioning is often utilized if bilateral blocks or catheters are to be performed preoperatively. A low-frequency curvilinear probe is positioned transverse in line with the second lumbar vertebra. Scanning may begin posterior to the midaxillary line to identify the internal oblique and transversus abdominis aponeuroses converging onto the transversalis fascia as the probe is translated posteriorly. The next muscle to be visualized is the QL muscle, which is bordered posteriorly by the erector spinae muscle group and anteriorly by the psoas major muscle. The needle trajectory is typically lateral to medial: for a lateral QL block, local anesthetic is injected at the lateral edge of the QL at the posterior origin of the TAP; for a posterior QL block, local anesthetic is injected in the fascial plane between QL and erector spinae muscles; and for an anterior QL block, the needle is inserted through the QL muscle to inject local anesthetic in the fascial plane between the QL and psoas major muscles.

#### Clinical pearls and evidence

The clinical features of the three QL blocks differ due to their injection locations, and it is beyond the scope of this article to delve into great detail about these differences. The anterior QL is unique compared to other abdominal fascial plane blocks due to the proximity of local anesthetic injection to the exiting roots of the lumbar plexus, which gives it the potential to provide visceral coverage but may also produce motor block. The current literature on QL blocks is heterogeneous but does demonstrate better pain scores and lower opioid consumption after abdominal surgery in patients who received QL blocks in addition to standard analgesia [51]. When looking specifically at anterior QL blocks in abdominal surgery, there are reported analgesic benefits over no block [52] Overall spread of injectate and area of clinical coverage are thought to be greater for QL block than the TAP block (Fig. 3). However, when compared to the much easier TAP block, the available data for anterior and other types of QL are equivocal and do not universally support QL block over TAP block in abdominal surgery [52].

Additionally, the anterior QL approach is a deep block, so anesthesiologists will need to assess coagulation status and potential bleeding risk [53]. TAP blocks may be more amenable to an anesthesiologist who is not an expert in regional anesthesia due to the ease of performance and safety profile, while QL blocks may be more likely to be performed at centers that specialize in advanced regional anesthesia techniques.

## Conclusion

Fascial plane blocks can provide additional pain relief for patients undergoing abdominal surgery as part of a multimodal analgesic regimen and are supported by guidelines. They can improve quality of recovery and reduce opioid consumption, pain-associated morbidity, and duration of hospital stay. Abdominal fascial plane blocks primarily provide somatic pain relief, and their targeted areas for coverage can be tailored to the locations of the primary surgical incision site(s). Our clinical decision-making tool complements existing guidelines and is designed for the general anesthesiologist. It simplifies the decision-making process for selecting an appropriate block and aims to increase access to regional analgesia for all patients who undergo abdominal surgery in alignment with the Plan A blocks framework.

## Data Availability

No datasets were generated or analysed during the current study.
